# Lipoic Acid Restores Binding of Zinc Ions to Human Serum Albumin

**DOI:** 10.3389/fchem.2022.942585

**Published:** 2022-07-11

**Authors:** Samah Al-Harthi, Kousik Chandra, Łukasz Jaremko

**Affiliations:** Smart Health Initiative (SHI) and Red Sea Research Center (RSRC), Bioscience Program, Biological and Environmental Science & Engineering (BESE), King Abdullah University of Science and Technology (KAUST), Thuwal, Saudi Arabia

**Keywords:** lipoic acid (LA), human serum albumin (HSA), zinc(II), fatty acids, zinc-binding, protein-ligand interactions, 2D methyl-TROSY, NMR

## Abstract

Human serum albumin (HSA) is the main zinc(II) carrier in blood plasma. The HSA site with the strongest affinity for zinc(II), multi-metal binding site A, is disrupted by the presence of fatty acids (FAs). Therefore, the FA concentration in the blood influences zinc distribution, which may affect both normal physiological processes and a range of diseases. Based on the current knowledge of HSA’s structure and its coordination chemistry with zinc(II), we investigated zinc interactions and the effect of various FAs, including lipoic acid (LA), on the protein structure, stability, and zinc(II) binding. We combined NMR experiments and isothermal titration calorimetry to examine zinc(II) binding to HSA at a sub-atomic level in a quantitative manner as well as the effect of FAs. Free HSA results indicate the existence of one high-affinity zinc(II) binding site and multiple low-affinity sites. Upon the binding of FAs to HSA, we observed a range of behaviors in terms of zinc(II) affinity, depending on the type of FA. With FAs that disrupt zinc binding, the addition of LA restores HSA’s affinity for zinc ions to the levels seen with free defatted HSA, indicating the possible mechanism of LA, which is effective in the treatment of diabetes and cardiovascular diseases.

## Introduction

Human serum albumin (HSA) accounts for over 60% (by mass) of human blood plasma proteins. It is considered the most abundant carrier protein in the human serum, with blood concentrations ranging from 30 to 50 g/L (∼0.53–0.75 mM), within a narrow pH range of 7.35–7.45 (Zhang et al., 2017; Al-Harthi et al., 2019). HSA is monomeric, globular, heart-shaped, and largely α-helical with some turns and extended loops, and contains 17 disulfide bridges shaping the protein fold, with one free cysteine at the 34th position (Cys 34) (He and Carter, 1992; Sugio et al., 1999; Bhattacharya et al., 2000; Handing et al., 2016). HSA consists of three topologically identical and structurally similar domains: domain I (aa 1–195), domain II (aa 196–383), and domain III (aa 384–585). Each domain comprises one antiparallel six-helix (sub-domain A) and one four-helix (sub-domain B) motif (Sugio et al., 1999). HSA possesses multiple ligand binding sites, in line with its extraordinary binding capacity with a wide range of ions and molecules (Stewart et al., 2003; Mauro et al., 2008; Bal et al., 2013). HSA plays a vital role in the regulation of blood metal ion homeostasis, including the transport and storage of transition metals. HSA is also responsible for binding, storing, and transporting numerous endogenous molecular ligands such as fatty acids (FAs), heme, and bilirubin, and exogenous ligands such as pharmacological drugs (Stewart et al., 2003; Bal et al., 2013; Fujiwara and Amisaki, 2013; Guizado, 2014).

HSA structures show the presence of two main ligand-binding sites with high affinity for diverse molecules referred to as “Sudlow’s sites” (I and II) (Sudlow et al., 1975). In addition, nine FA binding sites have been identified with different affinities (Fujiwara and Amisaki, 2013), and four metal-binding sites with varying metal ion specificities (Bal et al., 2013). Despite the substantial diversity of high-, medium- and low-affinity ligands, only two well-defined and distinct spatial conformations of HSA have so far been documented: one for defatted HSA (He and Carter, 1992; Sugio et al., 1999) and one for fatted HSA (Curry et al., 1998; Bhattacharya et al., 2000; Petitpas et al., 2001). Due to low solubility in water, FAs in blood plasma bind to HSA and are transported to other tissues (Lafontan and Langin, 2009; van der Vusse, 2009). Under normal physiological conditions, FAs are considered to be the primary physiological ligands of HSA. Most FAs in the blood plasma are bound to HSA (0.1–2.0 molar equivalents), and their binding affinities (K_D_) are chain length-dependent, ranging from 1.5 to 35 nM (Fanali et al., 2012; Friedrichs and Peters, 1997). Under elevated FA conditions (such as during intense exercise, fasting, or pathological conditions such as diabetes), more than 2 molar equivalents (up to 6 mol equiv.) of FAs are bound to HSA (Richieri and Kleinfeld, 1995). High levels of FAs in blood plasma are associated with a variety of diseases, including cancer, diabetes, and obesity (Richieri and Kleinfeld, 1995). Since FAs and ligands co-exist, ligand-metal binding affinities are modulated by the presence of FAs (Al-Harthi et al., 2019).

Among the ligands, the zinc ion is the second most abundant d-block metal ion within the human body and an essential micronutrient for multiple aspects of human metabolism (Saper and Rash, 2009), (Coverdale et al., 2018). Zinc is required for cell development and function. It plays a vital catalytic and structural role in different proteins such as zinc finger domains, and it is a component of catalytic centers in some enzymes (Bal et al., 2013). The free zinc ion Zn(II) is considered to be toxic to cells at high concentrations (Bozym et al., 2010). Therefore, cellular Zn(II) homeostasis is mostly tightly controlled (King et al., 2000). HSA is considered to be the main carrier of Zn(II) in plasma (whole blood concentration 19 µM (Report of the Task Group on Reference Man, 1979), with 75%–90% of Zn(II) in blood bound to HSA (Giroux and Henkin, 1973; Friedrichs and Peters, 1997; Blindauer et al., 2009). Stewart et al. (2003) estimated that ∼2% of circulating albumin molecules carry a zinc ion and that the modulation of their mutual affinity might have significant consequences (Barnett et al., 2013). Albumin transports newly-absorbed Zn(II) to the liver (Smith et al., 1979), and facilitates its uptake by endothelial cells (Rowe and Bobilya, 2000) and erythrocytes (Gálvez et al., 2001).

Despite the important role of HSA in zinc transport, the structure and location of the zinc-binding site in albumin have only recently been identified (Handing et al., 2016). The first X-ray crystallography data of Zn(II)- HSA binding at the atomic level was published in 2016 (PDB: 5IJF) (Handing et al., 2016). The crystal structure of the defatted HSA-Zn(II) complex, at pH 9, showed only one strong binding site (multi-metal binding site A, MBS-A), and up to nine secondary binding sites with a low metal affinity. Unfortunately, the resolution of the structure (refined at 2.65 Å) did not allow us to pinpoint their exact locations. Even isothermal titration calorimetry (ITC) showed only two Zn(II) binding sites in wild-type HSA (MBS-A, and one of the secondary sites) (Stewart et al., 2003; Handing et al., 2016).

Physiologically, HSA is not defatted, and it has a high-affinity FA binding site that can bind with FA at the ratio of 1:1 (Bhattacharya et al., 2000). The binding of Zn(II) to MBS-A is modulated by the binding of FAs at FA-binding site 2 because both sites lie at the interface between domain I and domain II of HSA. Zn(II) can only bind to MBS-A in the absence of FAs. When the level of FAs is elevated during intense exercise, fasting, or pathological conditions such as diabetes, it will disrupt zinc binding. As a result, zinc will be released from the site (Barnett et al., 2013). An increase cation in free Zn(II) concentration in blood has three major consequences, 1) redistribution of Zn(II) to other proteins, 2) interactions of Zn(II) with transporter proteins facilitating their export from plasma (Barnett et al., 2013), and 3) binding of zinc to cell-surface receptors and modulation of cell function (Zhu et al., 2017; Hershfinkel, 2018). Many studies have found correlations between diseases characterized by elevated FAs such as diabetes (Hawkins et al., 2003; Salgin et al., 2012) and cardiovascular disease (Little et al., 2010; Handing et al., 2016), and low plasma zinc concentrations. Here, we examined the interactions of zinc with HSA and the effect of different FAs on zinc binding to gain insights into the interplay between short, long and medium carbon tail length FAs and Zn(II).

## Materials and Methods

### Protein Preparation

De-fatted human serum albumin (HSA) lyophilized powder was purchased from Sigma-Aldrich (product no. A3782-1G; >99% pure base on gel electrophoresis). HSA was dissolved in 50 mM TRIS·HCl and 140 mM sodium chloride (NaCl) at pH 7.4 to obtain the required concentration for each experiment. The exact HSA concentration was determined by UV absorbance using NanoDrop One spectrophotometer using the molar extinction coefficient of 35,700 M^−1^cm^−1^ at 280 nm. The protein was then dialyzed overnight at 4°C against the respective buffer to remove impurities.

### Preparation of Fatty Acid/Human Serum Albumin Complexes

Fatty acids (FAs, Supplementary Table S1) were dissolved in 50% ethanol/water (*v/v*) and heated up to 70°C to obtain clear stock solutions with a concentration of 10 mM prior to each experiment. Solutions were diluted with TRIS-NaCl buffer to obtain 1 mM concentration and warmed up to about 50°C to facilitate FA dispersion. Then, the solutions were allowed to evaporate at room temperature and slightly cool down, and degassed for 20 min to eliminate alcohol before adding HSA. HSA-FA complexes were prepared by the addition of the appropriate volumes of FA stock to constant protein volume to obtain the desired FA:HSA molar ratios of 1:1, 2:1, 4:1, and 8:1. The mix was incubated with 500 rpm shaking for 2 h at 30°C to enable FA binding to HSA. Monitoring by 1D ^1^H zgesp spectra, we confirmed the presence of alcohol in the FAs was less than 0.5% by comparing to 5% alcohol sample that we supposedly started with, but due to our processing of the sample, most of the alcohol was evaporated. Supplementary Figure S1 shows a quality check if the overall processes affected the protein availability and activity.

### Isothermal Titration Calorimetry

90 µM of HSA from 0.7 mM stock was obtained for the measurements. ZnCl_2_ was prepared by dissolving the measured amount of ZnCl_2_ using the reaction buffer to obtain 2.7 mM concentration. Calorimetric experiments were performed using a Microcal PEAQ ITC (Malvern) at 25°C. Samples were degassed for 15 min before being loaded into ITC. 2.7 mM of ZnCl_2_ in the injection syringe was titrated into 90 µM of HSA (fatted and defatted) in the cell and continuously stirred at 750 rpm to ensure rapid mixing. The parameters for all experiments were set to one injection of 0.4 µL over 0.8 s followed by 54 injections of 0.7 µL over 1 s with an interval of 150 s between injections. An identical blank titration was performed to account for heat of dilution consisting of reaction buffer in the cell titrated by ZnCl_2,_ which was then subtracted from the main experiments. The data were processed and fitted using Microcal PEAQ-ITC analysis software provided with the instrument. The data were fitted to “Two sets of sites” fitting model assuming the protein has two non-identical sites for Zn(II) binding. Reported values of dissociation constants and their S.D. errors are the weighted averages from the triple replicate measurements.

### Nuclear Magnetic Resonance

Sample preparation for Zn(II) titration experiment: The Nuclear Magnetic Resonance (NMR) buffer used was 50 mM TRIS and 140 mM sodium chloride (NaCl) in 99% deuterium oxide at pH 7.4. HSA was dissolved in the NMR buffer to obtain a concentration of 2 mM. The titration of Zn(II) was performed using a stock solution of 40 mM zinc chloride (ZnCl_2_) dissolved in the NMR buffer. Aliquots of the stock solution were added to 2 mM HSA solution to obtain [ZnCl_2_]/[HSA] ratios of 0.1, 0.2, 0.6, 0.8, 1, 1.2, 1.4, 1.6, 1.8, 2, 2.5, 3, 3.5, 4, 6, and 8.

Sample preparation for competitive binding experiment: The HSA/FA complexes were prepared by dissolving HSA in the NMR buffer with the addition of a 1:6 ratio of palmitic acid, alpha-lipoic acid (ALA), dihydrolipoic acid (DHLA), a mixture of palmitic acid, and ALA, and a mixture of palmitic acid and DHLA acid to obtain HSA concentration of 100 µM. HSA/FA complexes were then incubated for 2 h and concentrated to 1 mM HSA/FA.

NMR data acquisition and processing: 2D [^1^H-^13^C] SOFAST methyl-TROSY experiment was recorded. NMR data were acquired at Bruker Neo 950, and 700 MHz spectrometers equipped with a cryoprobe and pulse filed gradients. Samples with a minimum of 500 µL were centered within the coils and maintained at 37°C (310 K). Spectra then were taken in the presence and absence of FAs. The 2D [^1^H-^13^C] SOFAST methyl-TROSY experiments were recorded. The major water peak was carefully suppressed by not exciting the peak and moving the offset to 1 ppm. For efficient excitation, a selective polychromatic pulse was applied in the methyl proton region (7.250 ms with a bandwidth of 2000 Hz). A total of 2,630 and 220 points were collected in the direct and indirect dimensions. We have recorded adequate points in the indirect dimension (here ^13^C) to ensure high resolution (47.79 Hz). The recycle delay was kept to 0.05 s. The data were zero-filled with 8,192 and 1,024 points and apodized before the FT in the second dimension. NMR data were processed with topspin version 4.0.7, and Sparky was used for further analysis. The detailed acquisition and processing parameters are given in Supplementary Table S2.

## Results

**FIGURE 1:**
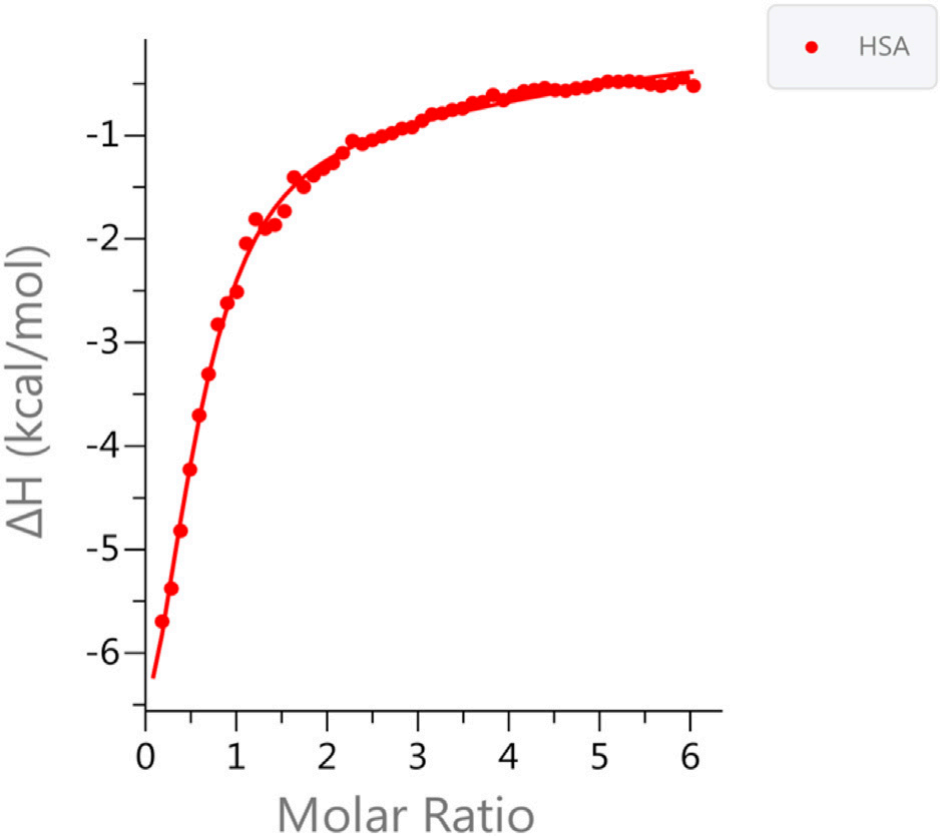
Measurements of Zn(II) binding affinity to HSA in the absence of FA. ITC data for Zn(II) binding to HSA in the absence of FA. The data was fitted to two set of binding sites. One high affinity site (MBS-A); K_D1_ = 5.43 ± 0.5 µM and up to seven secondary low affinity sites K_D2_ = 80 ± 5 µM was obtained.

**FIGURE 2:**
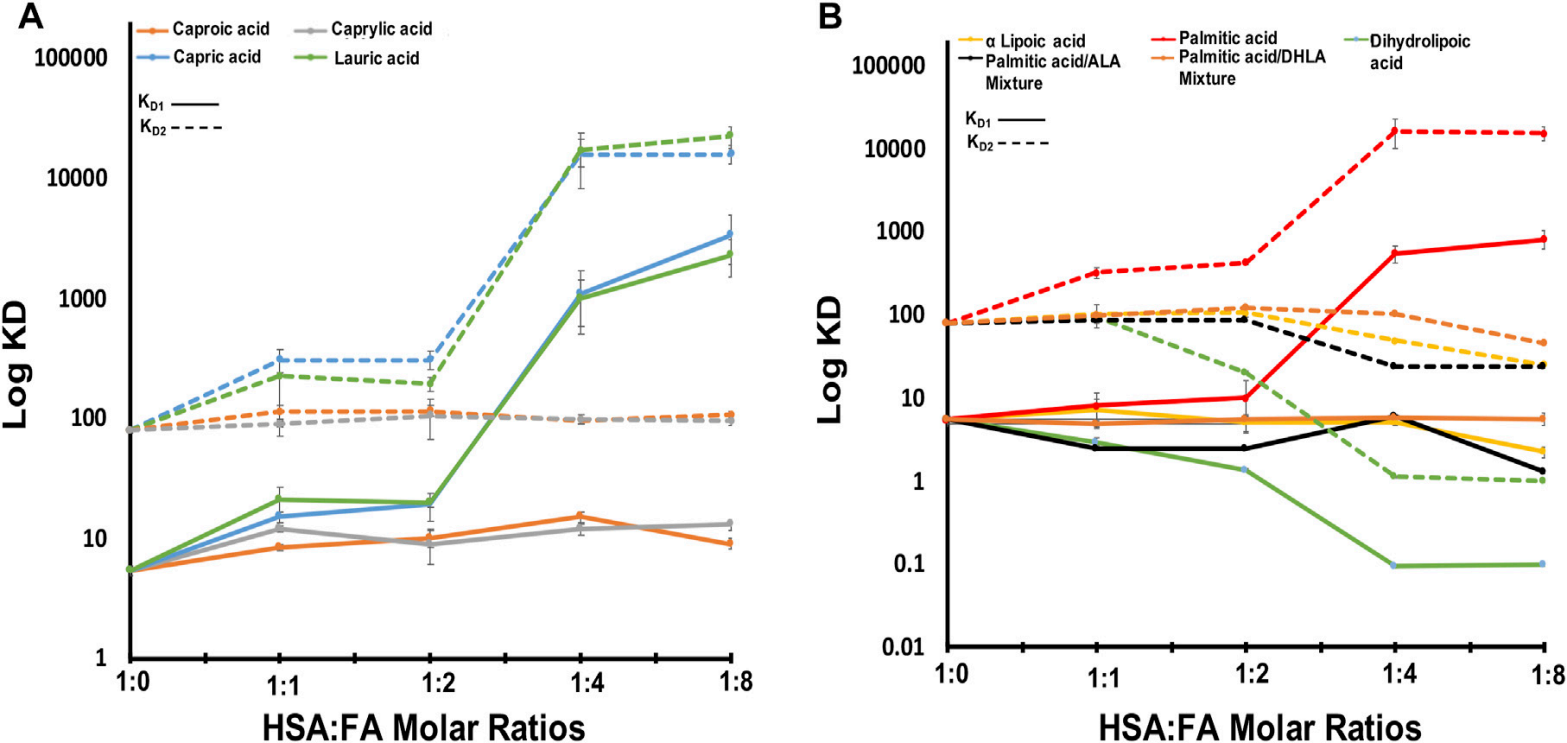
The obtained KD1,2 of HSA-FAs complexes compared to free HSA. **(A)** The KD values of HSA in complex with caproic acid, capric acid, caprylic acid, and lauric acid. **(B)** The KD values of HSA in complex with α lipoic acid, palmitic acid, dihydrolipic acid, palmitic and α lipoic acid mixture, and palmitic and dihydrolipic acid mixture. KD1 is indicated with solid lines and KD2 is indicated with dotted lines. Error bars represent standard deviation (SD).

We used ITC to examine the binding of zinc ions to HSA at physiological pH (pH 7.4) in the presence and absence of FAs. The data fit well with the “Two sets of sites” fitting model, in line with previous studies that reported the existence of more than one zinc site on HSA with different K_D_ values (Handing et al., 2016). The results show that the zinc ion binds to HSA in the absence of FAs, with high affinity (K_D1_ = 5.43 ± 0.5 µM). There is one additional set of binding Zn(II) sites comprising up to seven secondary sites with low affinity (K_D2_ = 80 ± 5 µM). The experimental data and the fitted example curve, is shown in Figure 1. After incubating six FAs with HSA, we used ITC to monitor the Zn(II) binding in selected different FA:protein ratios in the presence of different FAs. The results obtained are shown in Figure 2. The detailed fitted values for all the experiments are provided in Supplementary Table S3. The overall trends of the binding affinity are given in Table 1. The key findings are explained below.

**TABLE 1:** Trends of the Zn^2+^ binding affinity to HSA in the presence of various fatty acids based on the obtained K_D_ values of ZnCl_2_ titration.

Name	K_D1_ Zn^2+^ binding affinity to HSA trend	K_D2_ Zn^2+^ binding affinity to HSA trend
HSA-caproic acid (C6:0)	No effect	No effect
HSA-caprylic acid (C8:0)	No effect	No effect
HSA-ALA (C8:0)	Increase	Increase
HSA-DHLA (C8:0 reduced)	Increase	Increase
HSA-capric acid (C10:0)	Decrease	Decrease
HSA-lauric acid (C12:0)	Decrease	Decrease
HSA-palmitic acid (C16:0)	Decrease	Decrease
HSA-PA-ALA (C16:0-C8:0)	No effect	Increase
HSA-PA-DHLA (C16:0-C8:0) reduced	No effect	No effect

## Short-Chain Fatty Acids do Not Affect the Structure and Functionality of Human Serum Albumin

First, we titrated Zn(II) in the presence of an increasing amount of short-chain FAs like caproic (C6) and caprylic (C8) acid (molar ratio of FA:HSA of 1, 2, 4, and 8) and observed no effect on zinc binding to HSA (Figure 2A). The experimental data and the fitted curve, is shown in Supplementary Figure S2. The K_D_ values in the presence of caproic and caprylic acid confirm a slight decrease in Zn(II) affinity to HSA upon binding of such FAs. The caproic (C6) and caprylic (C8) acids binding to HSA did not significantly impact Zn(II) affinity rather, they decreased slightly (Figure 2A; 8% decrease observed for both sets of sites). We suggest that C6 and C8 did not induce a conformational change upon binding; therefore, there was no substantial effect on Zn(II) affinity. This is consistent with the study reported by *Lu et al.*; which showed that FAs with a chain length of less than ten carbon atoms are too short to induce a conformational change upon binding. Therefore, they cannot cause the allosteric switch required for zinc ions and FA crosstalk (Lu et al., 2012).

## Long-Chain Fatty Acids Decrease the Binding of Zinc Ions to Human Serum Albumin

**FIGURE 3:**
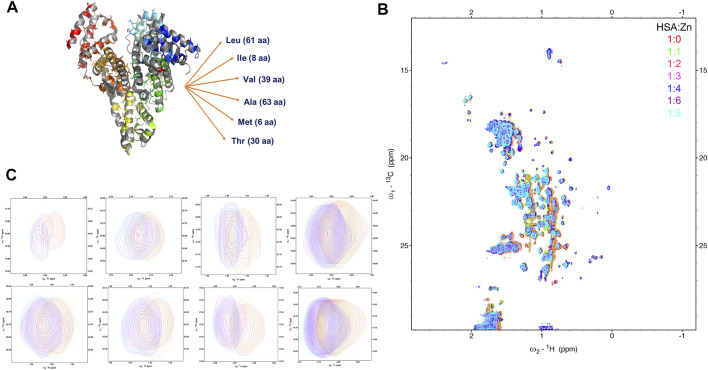
Monitoring the Zn(II) ions binding to HSA with natural abundance of ^13^C at atomic resolution. The 2D [^1^H-^13^C] SOFAST methyl-TROSY spectrum of HSA titrated with increasing ratio of Zn(II). **(A)** Structure of HSA in Zn(II) bound state highlighting 207 Methyl groups on six amino acids side chains LIVAMT. **(B)** the 2D Methyl-TROSY spectrum of free HSA (red) overlaid with HSA titrated with different ratios of ZnCl_2_ (1:1 (green), 1:2 (pink), 1:3 (magenta),1:4 (blue), 1:6 (purple) and 1:8 (cyan)). **(C)** Selected peaks showing different behaviors of signals.

In the presence of long-chain FAs like capric (C10), lauric (C12), and palmitic (C16) acid (Figures 2A,B), we observed a dramatic decrease in Zn(II) affinity as the molar ratio increased. The experimental data and the fitted curve, is shown in Supplementary Figure S3. Zn(II) affinity was slightly (∼10%) affected by the first two ratios (1:1 and 2:1 FA:HSA) (Figure 2), suggesting that FAs did not fully occupy FA-binding site 2 (FA2). Hence, Zn ions can still bind. Upon shifting to higher ratios (4:1 and 8:1), Zn(II) affinity decreased dramatically (>80%), indicating FA2 was fully populated, preventing Zn(II) binding. This disruption of MBS-A can be explained by the structural alignment of HSA in a Zn-bound state and HSA in FA-bound form (Coverdale et al., 2018). Proposed structural rearrangement upon long-chain FA binding to HSA is in line with large changes on the high resolution 2D [^1^H-^13^C] SOFAST methyl-TROSY spectra, that probe the chemical environments of mehyl groups of HSA (Figure 3B; Supplementary Figure S4). FA binding requires changes in the domain I and II interface, and that causes a relative movement of sidechain nitrogen of His67 by 7.5 Å, which was in direct coordination with the metal ion (Coverdale et al., 2018).

## Lipoic Acid in Both Reduced and Oxidized Form Rescues the Human Serum Albumin Zinc-Binding

Lipoic acid (LA, reduced and oxidized forms) is one of the most powerful biological antioxidants with high efficacy found naturally in our diet. It is involved in the chelation of metal ions, regeneration of antioxidants, repair of oxidatively damaged proteins, and under physiological conditions exists as a cofactor for many enzymes. Its strong antioxidant activity made it a prominent therapeutic agent that is used in the treatment of many diseases, such as diabetes, cardiovascular diseases, cancer, and AIDS. Here we incubated HSA with alpha-lipoic acid (ALA, oxidized LA) or dihydrolipoic acid (DHLA, reduced LA) and titrated it with Zn(II) (Figure 2B). The experimental data and the fitted curve, is shown in Supplementary Figure S5. At a low molar ratio (1:1 and 2:1, FA:HSA), the binding of ALA to HSA did not affect the affinity for Zn(II), which remained similar to that of free HSA (Figure 2B). However, at higher molar ratios of 4:1 and 8:1, the affinity increased by almost 37% and 60%, respectively, for both sets of sites. Binding of DHLA to HSA at 1:1 molar ratio increased the Zn(II) affinity dramatically and continued to increase it at ratios of 2:1 and 4:1 for both sets of sites. This increase in Zn(II) affinity might be due to ALA antioxidant activity. A 1D NMR study that examined ALA binding to bovine serum albumin (BSA) reported evidence of strong hydrophobic interaction and binding of ALA to BSA (Lescanne et al., 2018). Also, the study demonstrates that ALA coats BSA providing a protective mechanism. Since there are structural and functional similarities between HSA and BSA (75% similarities in terms of ligand-binding affinity), there is a possibility that ALA redox chemistry and protective mechanism increase the amount of healthy (native) HSA. Native HSA is present primarily in reduced form, although some oxidized fractions can exist under physiological conditions. Hence, persevering its major functions and increasing Zn(II) binding affinity. Alternatively, the binding of ALA induces structural rearrangements that may expose major Zn(II) binding sites making them more active.

We further examined the effect of two FA mixtures on zinc binding to albumin. With a mixture of palmitic acid and ALA, K_D1_ showed a constant value suggesting no effect on zinc binding to HSA (Figure 2B). The experimental data and the fitted example curve, is shown in Supplementary Figure S6. In this case, ALA did not enhance zinc binding. However, it protected zinc high-affinity sites from palmitic acid, which can disrupt the zinc-binding sites. Beyond FA:HSA 1:1 ratio, we saw a decrease in the K_D2_ values suggesting that ALA binds stronger than palmitic acid. With a mixture of palmitic acid and DHLA, both K_D1_ and K_D2_ showed a constant value in the presence of an increasing amount of the mixture (Figure 2B). As for ALA, DHLA did not enhance zinc-binding, but it provided protection of zinc affinity sites from disruption by palmitic acid. Therefore, LA may modulate the architectures of both fatted and defatted HSA, altering zinc binding.

### Lipoic Acid Modulates the Architecture of Human Serum Albumin

To understand the conformational rearrangement and structural features further, we performed NMR measurements. We monitored Zn(II) binding to HSA by recording a series of 2D [^1^H-^13^C] SOFAST methyl-TROSY experiments with the addition of ZnCl_2_ in the titration experiment. This recently developed method allows rapid recording of high-quality methyl ^1^H-^13^C correlation spectra of a protein with high molecular weight with natural abundance of NMR-active ^13^C isotope in a highly sensitive manner. Methyl groups are used as spectroscopic probes in this case. Here, we aimed to detect the resulting conformational change due to Zn(II) binding to HSA. We are recording these spectra focusing on methyl groups of HSA using them as natural abundance isotopes. HSA contains six amino acids that have methyl groups in their sidechains that are highlighted in Figure 3A, LIVATM (Leu, Ile, Val, Ala, Thr, and Met). The movements of those methyl groups could indicate a conformational change due to Zn(II) binding. Figure 3B shows the full HSA spectrum in apo form overlayed with HSA that was titrated with different ratios of ZnCl_2_. Small local chemical shifts for some residues were noted, indicating little or no effect on the overall HSA 3D structure. Some resonances remained in their native region, such as methionine signals that displayed no movement even with an increasing amount of Zn(II). Others were unaffected by the addition of Zn(II) initially. Upon increasing the amount of zinc above a 4:1 (Zn(II):HSA) molar ratio, the behavior of some signals coming from those resonances was moderately affected. For instance, one signal of alanine was closely inspected, and with the addition of an excess of Zn(II) the signal started to show some small distinct chemical shift. Selective broadening was observed for some resonances. Some resonances were lost mainly at 8:1 molar ratio, indicating an increase in conformational dynamics and mutual interconversion in the NMR timescale (Figure 3C shows selected signals).

**FIGURE 4:**
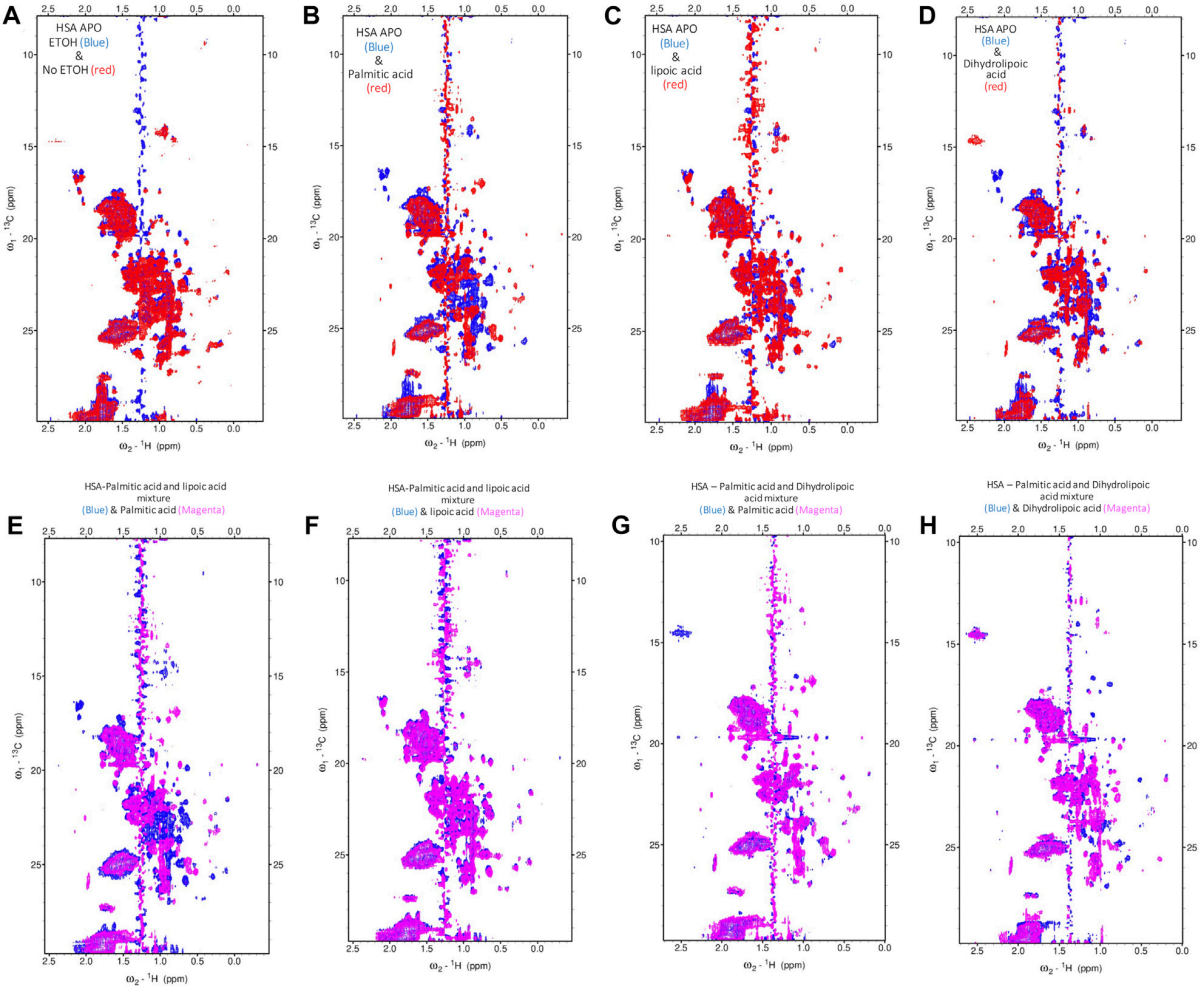
Probing the conformational changes of HSA with high-resolution NMR. The 2D [^1^H-^13^C] SOFAST methyl**-**TROSY spectra. **(A)** Free-HSA spectrum with 1% (v/v) ETOH (blue) overlaid with free HSA without ETOH (red). **(B)** Free-HSA spectrum with ETOH (blue) overlaid with palmitic acid (red). **(C)** Free-HSA spectrum with ETOH (blue) overlaid with lipoic acid (red). **(D)** Free-HSA spectrum with ETOH (blue) overlaid with dihydrolipoic acid (red). **(E)** HSA-palmitic acid and lipoic acid mixture (blue) overlaid with palmitic acid (magenta). **(F)** HSA-palmitic acid and lipoic acid mixture (blue) overlaid with lipoic acid (magenta). **(G)** HSA-palmitic acid and dihydrolipoic acid mixture (blue) overlaid with palmitic acid (magenta). **(H)** HSA-palmitic acid and dihydrolipoic acid mixture (blue) overlaid with dihydrolipoic acid (magenta).

To fully understand the competitive FA binding to HSA observed by ITC, we monitored HSA upon the addition of a 6:1 (FA:HSA) molar ratio of a mixture of palmitic acid and ALA, and a mixture of palmitic acid and DHLA by recording 2D [^1^H-^13^C] SOFAST methyl-TROSY spectra (Figure 4; Supplementary Figure S2). The addition of palmitic acid to HSA induced a specific conformational change in the albumin as many residues were broadened out (Figure 4B). The broadening of the signals represents a less structured conformation where palmitic acids destabilize the FA binding sites. The addition of ALA to HSA did not significantly alter the original conformation (Figure 4C); however, the addition of DHLA induced a different conformational change in albumin that might cause Zn(II) binding sites to be exposed (Figure 4D). The spectrum of palmitic and ALA mixture overlaid with palmitic acid alone (Figure 4E) or ALA alone (Figure 4F) showed that ALA binds stronger than palmitic acid to HSA, indicating that ALA restores the HSA to its original conformation. Similarly, the spectrum of palmitic and DHLA mixture overlaid with palmitic acid alone (Figure 4G) or DHLA alone (Figure 4H) showed that DHLA binds stronger than palmitic acid to HSA. The conformational change induced upon the binding of DHLA provides a protection mechanism from palmitic acid. From the above description, it is evident that LA, in both oxidized and reduced forms, modulates the architectures of both fatted and defatted HSA.

## Discussion

### Zinc Binding Capacity to Human Serum Albumin

Zn(II) is an essential metal ion for the activity of multiple enzymes and transcription factors. Among many other transporting proteins, albumin is the main Zn(II) carrier (75%–90% of plasma Zn(II) is bound to albumin) found in the blood plasma. Thus, albumin controls the free Zn(II) concentration that is available to cells through membrane transporters or by other proteins (Handing et al., 2016). Albumin itself has been the subject of many studies for years. However, inside cells, albumin exists with different FAs. The binding of Zn(II) to site MBS-A is modulated by the binding of FAs at FA2 because both sites lie at the interface between domain I and domain II of HSA and require interactions with residues from both domains (His67 and Asn99 from domain I and His246 and Asp249 from domain II). Thus, the binding of FAs and Zn(II) are not independent and found to be linked and Zn(II) can only bind to site MBS-A on albumin in the absence of FAs. Under conditions where the FAs levels are elevated, the zinc binding will be disrupted and Zn(II) is released. Here, we attempted to investigate Zn(II) binding to HSA by means of ITC and NMR and, most importantly, how different FAs modulate the binding.

### The Presence of Multiple Zinc-Binding Sites on Human Serum Albumin

Based on ITC data, free HSA has one high-affinity site, referred to as MBS-A, and up to seven secondary low-affinity sites (K_D1_ = 5.43 ± 0.5 µM and K_D2_ = 80 ± 5 μM, respectively). The presence of those secondary sites can extend the zinc-binding capacity of HSA, and this is significant under conditions where Zn(II) concentration is elevated or MBS-A is disrupted by FA binding. Thus, those sites can bind to zinc ions, controlling its free concentration in the blood. Our results are consistent with a previously published ITC study demonstrating the presence of two sites with significant affinity (K_D1_ = 1.7 ± 0.3 µM and K_D2_ = 65 ± 21 µM) (Handing et al., 2016). However, the results of this study were fitted using “two sequential sites”. In our study, three possible models were explored to fit the data, and we found a “Two sets of sites” model has the best goodness-of-fit according to the reported reduced Chi-seq values for apo HSA and HSA-FA complex (C6:0). Based on the NMR titration, we could say that Zn(II) prefers parallel binding rather than sequential binding, as the minor chemical shifts happen uniformly after adding the metal ion. We did not observe in any specific spectral region chemical shift change or appearance of new signals happening first, followed by others.

### Conformational Rearrangement Upon Zn(II) Binding

**FIGURE 5:**
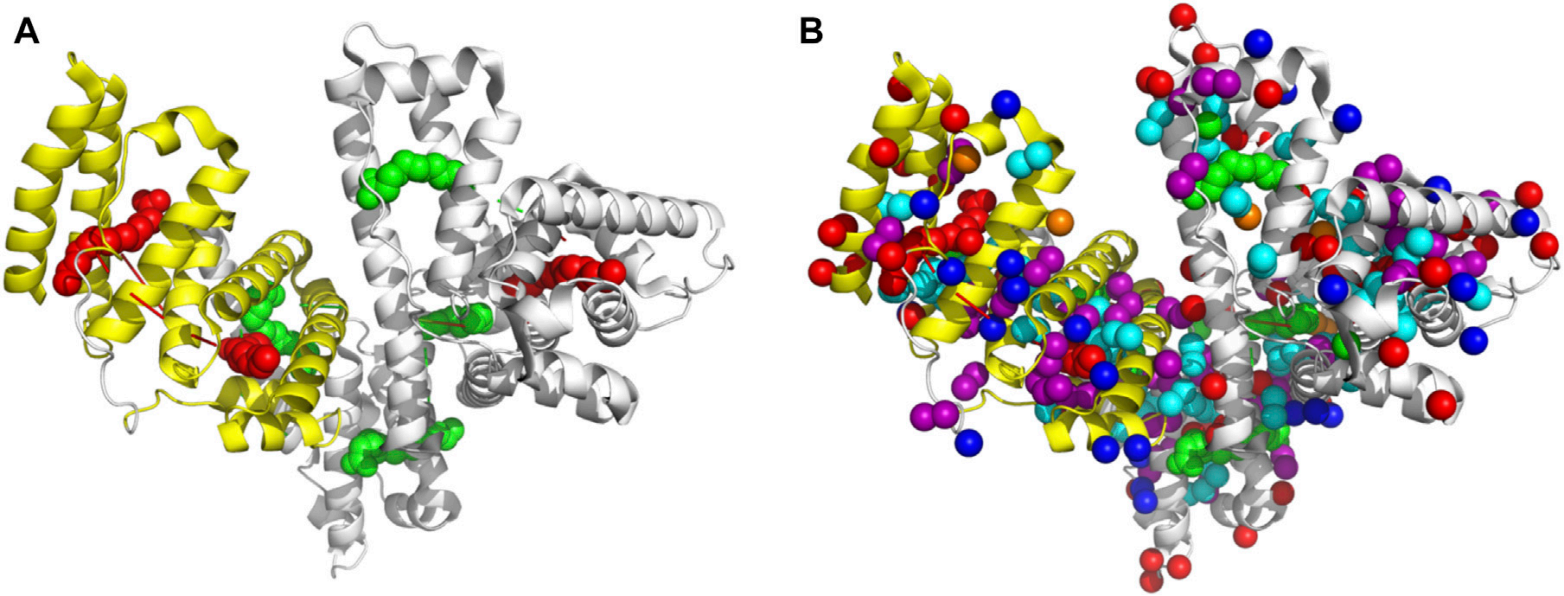
Figure **(A)** reperents HSA structure bound with hexadecanoic acid or palmitic acid (PDB ID: 1E7H). The high-affinity (red) sites and low-affinity sites are highlighted (green). In figure **(B)**, the Methyl groups are highlighted.

The 2D [^1^H-^13^C] SOFAST methyl-TROSY experiment allowed us to obtain a high-quality methyl ^1^H-^13^C correlation spectrum by rapidly tracking methyl resonances of unlabeled proteins. HSA with a molecular weight of 66.5 kDa contains 207 methyl groups (Figure 3A) on six amino acid sidechain types (Leu-61, Ile-8, Val-39, Ala-63, Thr-30, and Met-6) that we used to allow the generation of the methyl map (Figure 3B). The methyl groups on HSA structure bound with palmitic acid (PDB ID: 1E7H) are shown in Figure 5. The high-affinity sites and low-affinity sites are highlighted in red and green, respectively. So far, HSA resonance assignments are not available; therefore, the exact localization of those methyl groups in the HSA 3D structure is unknown in the 2D HSQC spectra. However, tracking the movements of those methyl groups upon the increasing amount of ZnCl_2_ could indicate that a conformational change has occurred due to Zn(II) binding. From the study by Blindauer (Coverdale et al., 2018), the sites with stong affinity sites for FA are very crucial. Among all the three strong affinity sites, we see a large populations of methyl groups aroubd, whose confirmation change can actually probe the metal binding. Overall, upon the addition of Zn(II), most of the signals remained constant. However, some signals displayed minor chemical shift perturbation, indicating a minor conformational change has been induced as a result of Zn(II) binding at a potential close by Zn(II) binding site. In fact, the methyl groups act as a probe for conformational change due to ligand binding in several structural studies (Wiesner and Sprangers, 2015; Lescanne et al., 2018). For more accurate information, further investigations are required using recombinant human albumin (rHSA) with labeled isotopes to pinpoint the exact locations of Zn(II) binding sites.

### Lipoic Acid Restores the Conformation and Zn-Binding of Human Serum Albumin

We observed that adding palmitic acid to HSA leads to many peak broadenings, which indicates a loosening of structure and an increase in conformational dynamics compared to the free state. This change in conformation is closely linked with the destruction of the Zn(II)-binding ability of the strong FA binding site. Thus a change in conformation is directly connected to the Zn(II)-binding ability, and the functionality of HSA concerning the regulation of metal ions in blood plasma. With the further addition of LA, either in oxidized or reduced form (ALA or DHLA, respectively), HSA is restored to its original conformation. This is evident from the overlay of the spectrum of palmitic acid and ALA mixture with palmitic acid alone (Figure 4E) or ALA alone (Figure 4F). From these, we can conclude that ALA binds stronger than palmitic acid to HSA, restoring HSA to its original conformation and Zn(II)-binding ability. We observed a similar scenario from the spectrum of palmitic acid and DHLA mixture overlaid with palmitic acid alone (Figure 4G) or DHLA alone (Figure 4H). In both cases, the conformational change induced upon the binding of palmitic acid is prevented, and the functionality in terms of Zn(II)-binding is restored. Recently, a great deal of attention has been given to the possible antioxidant functions of LA (reduced or oxidized forms). Mainly due to the antioxidant properties of these compounds and their preventive and therapeutic implications and applications. Previously we have shown that Zn(II) affinity is increased by ALA alone. A 1D NMR study examining ALA binding to bovine serum albumin (BSA) reported strong hydrophobic interaction and binding (Schepkin et al., 1994). Since there are structural similarities between HSA and BSA, there is a possibility that ALA increases the amount of healthy (native) HSA in the reduced form, which may be responsible for increased Zn(II) binding affinity.

The findings of this study can be helpful in understanding the contributing role of LA in Zn(II)-binding to HSA. Our results can offer future perspectives for investigations of the use of LA in the food and pharmaceutical industries as a dietary intervention and apply HSA as an efficient delivery system for LA. Having in mind that LA is a very potent antioxidant and its use can alleviate a number of conditions related to oxidative stress, it is essential to understand and obtain a detailed analysis of LA mode of interaction with HSA, a universal transporter in the plasma, as the properties of this interaction, are still unknown and undefined.

## Conclusion

Zinc distribution and delivery to cells is tightly controlled. Serum albumin is the major carrier of Zn(II) throughout the blood plasma, but it also serves as a transporter of other physiologically important molecules such as FAs. HSA has a strong binding site for Zn(II) (Handing et al., 2016) that overlaps with FA binding site 2 (Coverdale et al., 2018), establishing an interplay between FAs and Zn(II). Even the physiologically relevant level of FAs can impact Zn(II) affinity to HSA, resulting in an imbalance of zinc distribution in blood plasma leading to a modulation of the function of many cell types. Here we investigated zinc ions interactions with HSA and the effect of different FAs using NMR and ITC, at atomic level resolution in a quantitative manner. We found HSA has one high-affinity metal-binding site and multiple low-affinity sites. Upon binding FAs to HSA, all kinds of modulations are possible. These can range from no effect to moderate to a significant increase or decrease depending on the type of FA. Importantly, LA completely restores the Zn(II)-binding ability, which is destroyed by palmitic acid. Our results show the importance of FA-modulated Zn(II) interaction as the key controlling factor for Zn(II) release inside cells.

## Data Availability

The original contributions presented in the study are included in the article/Supplementary Material, further inquiries can be directed to the corresponding author.
