# Supplementary material for: Lipoic Acid Restores Binding of Zinc Ions to Human Serum Albumin
Source: Front Chem. 2022 Jul 11;10:942585. doi: 10.3389/fchem.2022.942585 (PMC9309503; doi:10.3389/fchem.2022.942585)
Supplement: Supplementary file 1 [file DataSheet1.PDF]

Supplementary materials

for

Lipoic Acid Restores Binding of Zinc Ions To Human Serum Albumin

Samah Al-Harthi[1], Kousik Chandra[1], Łukasz Jaremko[1]*

[1]*Smart Health Initiative (SHI) and Red Sea Research Center (RSRC), Bioscience Program, King Abdullah University of Science and Technology (KAUST), Biological and Environmental Science & Engineering (BESE), 23955-6900 Thuwal, Saudi Arabia*

* Correspondence:
Lukasz Jaremko
e-mail: lukasz.jaremko@kaust.edu.sa

Keywords: lipoic acid (LA), human serum albumin (HSA), zinc(II), fatty acids, zinc-binding, protein-ligand interactions, 2D [^{1}H-^{13}C] SOFAST methyl-TROSY, NMR

Emails of all co-authors:

Samah Al-Harthi: samah.harthi@kaust.edu.sa

Kousik Chandra: kousik.chandra@kaust.edu.sa

Lukasz Jaremko: lukasz.jaremko@kaust.edu.sa

Fatty acid common name	Fatty acid systematic name	Chemical formula	Lipid number	Molecular weight of FA (g/mol)	Structure
N-caproic acid	Hexanoic acid	$C_6H_{12}O_2$	C6:0	116.16	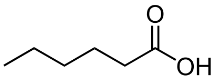
Caprylic acid	Octatonic acid	$C_8H_{16}O_2$	C8:0	144.21	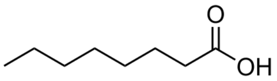
L- α-lipoic acid	Lipoic acid	$C_8H_{14}O_2S_2$	C8:0	206.32	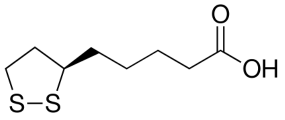
(R)-Dihydrolipoic acid	Reduced lipoic acid	$C_8H_{16}O_2S_2$	C8:0	208.3	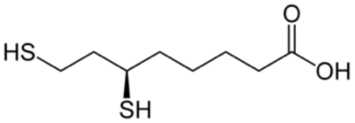
Capric acid	Decanoic acid	$C_{10}H_{20}O_2$	C10:0	172.27	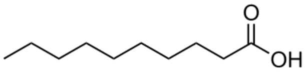
Lauric acid	Dodecanoic acid	$C_{12}H_{24}O_2$	C12:0	200.32	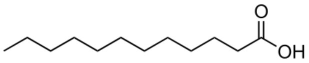
Palmitic acid	Hexadecanoic acid	$C_{16}H_{32}O_2$	C16:0	256.43	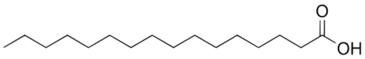

Table S1: List of fatty acids selected for the current project. The table shows the common and systematic names of the fatty acids selected for this experiment along with the chemical formula and molecular weight.

Pulse program	F1	F2	No of scans	TD	Acquisition Time (s)	Resolution (Hz)
Methyl SOFAST-HMQC	^{13}C	^{1}H	256	F1 = 220<br>F2 = 2630	F1 = 0.02<br>F2 = 0.09	F1 = 47.79<br>F2 = 10.56

Table S2: The experimental parameters for 2D [^{1}H-^{13}C] SO FAST methyl-TROSY.

Compound	K_{D1}		K_{D2}	
HSA Apo	5.4 ± 0.5 µM		80 ± 5 µM	
HSA-caproic acid (C6:0)	1:1	6.5 ± 1.3 µM	1:1	97 ± 15 µM
	1:2	6 ± 1.6 µM	1:2	88 ± 11 µM
	1:4	7 ± 1.7µM	1:4	96 ± 6 µM
	1:8	7.3 ± 0.9 µM	1:8	102 ± 8 µM
HSA-caprylic acid (C8:0)	1:1	6 ± 0.7 µM	1:1	87 ± 6 µM
	1:2	8.3 ± 1.8 µM	1:2	91 ± 7 µM
	1:4	8.7 ± 1.4 µM	1:4	98 ± 9 µM
	1:8	9 ± 1.5 µM	1:8	95 ± 5 µM
HSA-α-lipoic acid (C8:0)	1:1	5 ± 1.8 µM	1:1	94 ± 8 µM
	1:2	5.4 ± 1.2 µM	1:2	90 ± 11 µM
	1:4	5 ± 0.4 µM	1:4	49 ± 3 µM
	1:8	2.2 ± 0.3 µM	1:8	25 ± 2.2 µM
HSA-capric acid (C10:0)	1:1	15 ± 1.6 µM	1:1	307 ± 6.6 µM
	1:2	19 ± 5 µM	1:2	309 ± 5.3 µM
	1:4	1.1 ± 0.5 mM	1:4	16 ± 7.7 mM
	1:8	3.4 ± 1.5 mM	1:8	16 ± 2.8 mM
HSA-lauric acid (C12:0)	1:1	21 ± 0.6 µM	1:1	195 ± 1.3 µM
	1:2	20 ± 1.9 µM	1:2	225 ± 2.9 µM
	1:4	1 ± 0.5 mM	1:4	17 ± 4.4 mM
	1:8	2.3 ± 0.7 mM	1:8	19.3 ± 4.4 mM
HSA-palmitic acid (C16:0)	1:1	8 ± 1.5 µM	1:1	318 ± 4.7 µM
	1:2	10 ± 1.3 µM	1:2	419 ± 2.6 µM
	1:4	542 ± 1.9 µM	1:4	16 ± 1.1 mM
	1:8	806 ± 2.3 µM	1:8	15 ± 2.9 mM
HSA-DHLA (C8:0 Reduced)	1:1	2.96 ± 0.3 µM	1:1	90.2 ± 1.5 µM
	1:2	1.34 ± 0.05 µM	1:2	20 ± 1 µM
	1:4	93.6 ± 4 nM	1:4	1.11 ± 0.08 µM
	1:8	99.0 ± 1 nM	1:8	1.0 ± 0.03 µM
HSA-PA-ALA (C16:0-C8:0)	1:1	6.98 ± 0.07 µM	1:1	71.4 ± 0.5 µM
	1:2	6.13 ± 1.4 µM	1:2	50 ± 7<br>1.7 µM
	1:4	5.34 ± 0.4 µM	1:4	34 ± 0.2 µM
	1:8	5 ± 0.2 µM	1:8	11 ± 0.4 µM

HSA-PA-DHLA (C16:0-C8:0) Reduced	1:1	4.9 ± 0.45 µM	1:1	97.1 ± 1.5 µM
	1:2	5.5 ± 0.63 µM	1:2	92 ± 0.7 µM
	1:4	5.8 ± 0.45 µM	1:4	88 ± 0.5 µM
	1:8	5.6 ± 0.95 µM	1:8	45 ± 1.2 µM

Table S3:List of obtained K_D values of $ZnCl_2$ titrations

List of obtained K_D values from tree replicates of $ZnCl_2$ titrations to HSA in the presence of six fatty acids with HSA:FAs ratio of 1:1, 1:2, 1:4, and 1:8.

Preparation of fatty acid/HSA complexes

SDS gel was done on 10 samples; HSA Apo, HSA incubated with zinc, HSA with FA:HSA ratio of 1:1, 2:1, 4:1 and 8:1 before incubation and after 2 hours incubation to check if any of the processes affected the albumin itself or caused dimerization and reduced the available active HSA for binding to either Zn(II) or FAs. As shown in the gel, about 98% of HSA protein were free monomers and approximately 1-2% was dimerized. Therefore, the overall processes did not affect the protein availability and activity with the exception of the N-terminal truncation which had no effect on our measurements.

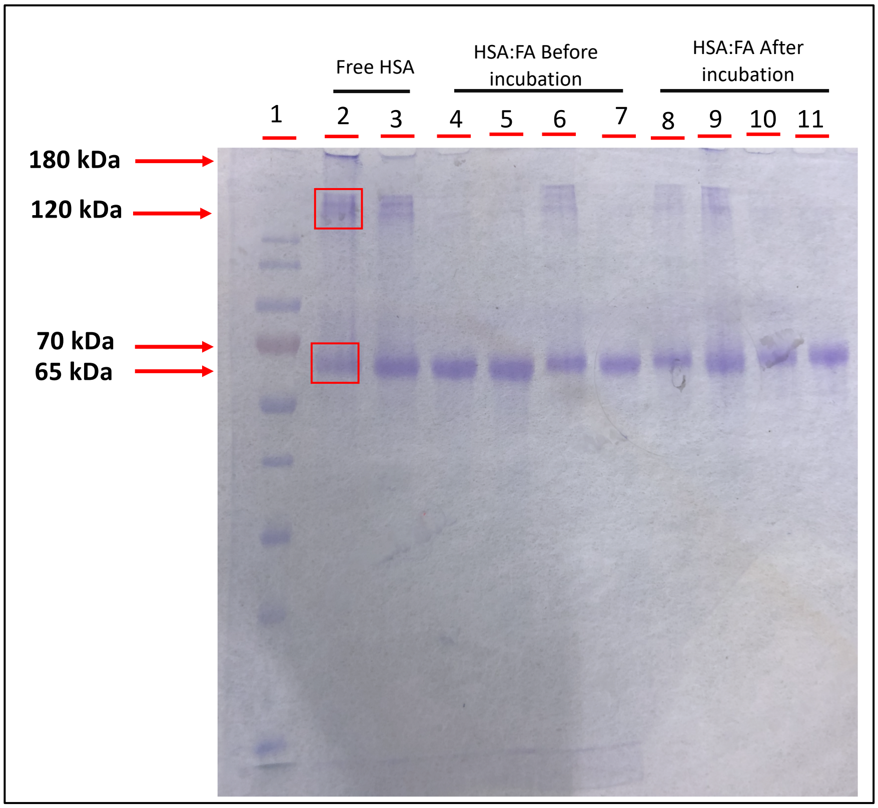

Figure S1:SDS page gel. Gel was run with HSA Apo (lane 2), HSA Apo with Zn (lane 3), FA:HSA ratio of 1:1, 2:1, 4:1 and 8:1 before incubation (lane 4, 5, 6, and 7 respectively) and after incubation (lane 8, 9, 10, and 11). Lane 1 is the molecular weight marker.

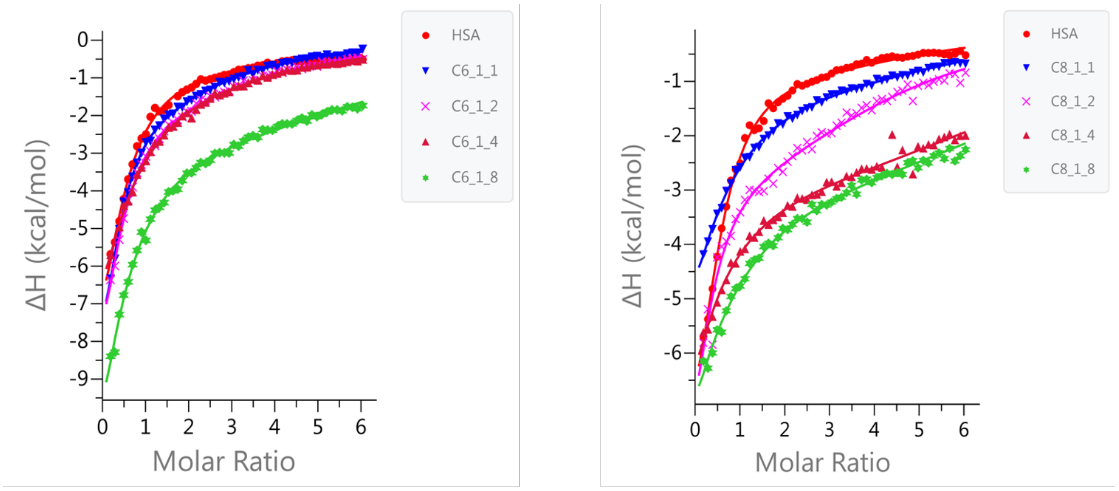

Caproic acid (C6) Caprylic acid (C8)

Figure S2: Measurements of Zn(II) binding affinity to HSA- FA complex. ITC data for Zn(II) binding to HSA- Caproic acid (C6) and HSA- Caprylic acid (C8).

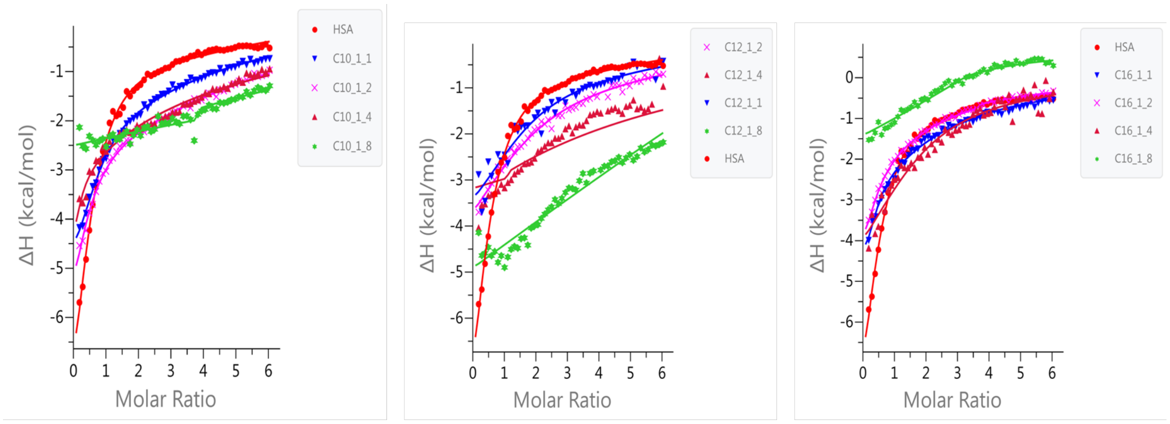

Capric acid (C10) Lauric acid (C12) Palmitic acid (C16)

Figure S3: Measurements of Zn(II) binding affinity to HSA- FA complex. ITC data for Zn(II) binding to HSA- Capric acid (C10), HSA- Lauric acid (C12) and HSA- Palmitic acid (C16)

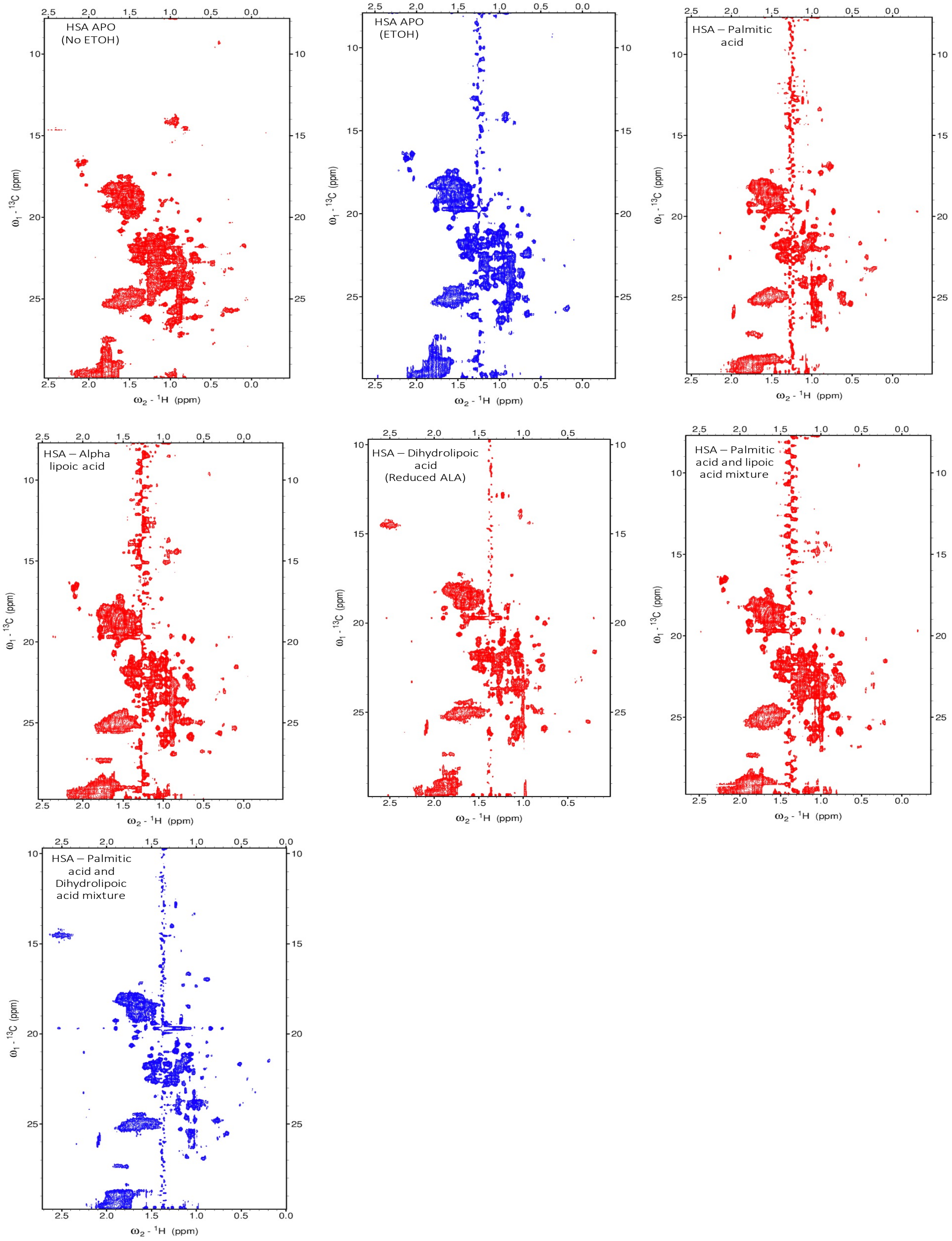

Figure S4: The 2D [^{1}H-^{13}C] methyl SO FAST-TROSY spectra of natural abundance HSA with various FAs and lipoic acid.

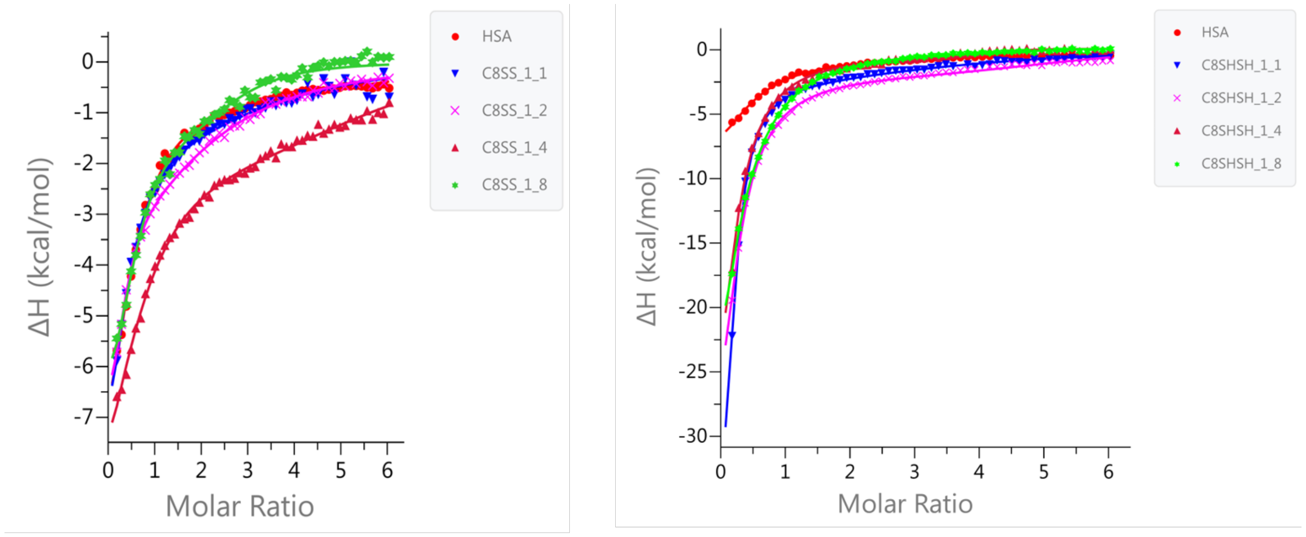

Alpha-lipoic acid(C8) Dihydrolipoic acid

Figure S5: Measurements of Zn(II) binding affinity to HSA- FA complex. ITC data for Zn(II) binding to HSA-Alpha-lipoic acid(C8), HSA- Dihydrolipoic acid

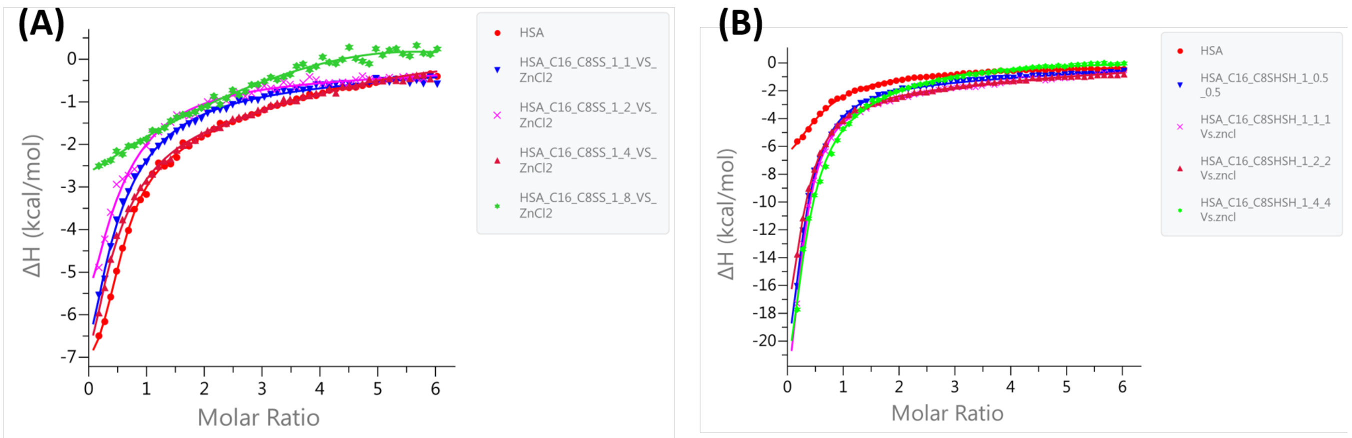

Figure S6: Measurements of Zn(II) binding affinity to HSA- FA mixture complex. ITC data for Zn(II) binding to (A) HSA-Palmitic acid and lipoic acid mixture. (B) HSA-Palmitic acid and Dihydrolipoic acid mixture. 90µM of protein was titrated with 0.7 µL injections of a 2.7 mM $ZnCl_2$ solution for 54 injections over 1 s with interval of 150 s between injections at 25 °C.
